# To Our Readers

**Published:** 1892-03-05

**Authors:** 


### To Our Readers.

Owing to a very general wish, we have determined to again enlarge The Hospital on and from the issue of April 2nd, 1892. It will then be more than double its original size. This will enable us to give new features, and to improve the appearance and style of the paper. We propose to devote a certain space each week to Institutional matters, including the Construction and Plans of new Hospitals and Institutions; the Relative Expenditure; the various improved methods which are introduced from time to time; the illustration of new Appliances which have an important bearing upon treatment or hygiene, or both; improvements in Furniture and Fittings; and an Enquiry and Answer Column dealing with all questions of Administration and Management. The circulation of The Hospital has steadily increased from the first, and now represents so many thousands of copies weekly that, having regard to the increased size and to the great demand, it has been decided to raise the price from One Penny to Twopence per copy on and after April 2nd next. Those of our readers who prefer to subscribe direct, by sending their subscriptions to the Publisher, at the Office, 140, Strand, W.C., will be supplied with The Hospital every week, post free, for 8s. 6d. a year; 5s. for tions will be payable in advance, and current subscribers will be supplied without additional charge for the unexpired term yet to run. The monthly parts will be issued at sixpence each, or post free for 7s. 6d. a year.

